# Computed tomography derived anatomical predictors of vascular access complications following transfemoral transcatheter aortic valve implantation: A systematic review

**DOI:** 10.1002/ccd.30918

**Published:** 2023-11-22

**Authors:** Vitaliy Androshchuk, Omar Chehab, Bernard Prendergast, Ronak Rajani, Tiffany Patterson, Simon Redwood

**Affiliations:** ^1^ School of Cardiovascular Medicine & Sciences, Faculty of Life Sciences & Medicine King's College London London UK; ^2^ Department of Cardiology St Thomas’ Hospital, King's College London London UK; ^3^ School of Biomedical Engineering and Imaging Sciences, Faculty of Life Sciences & Medicine King's College London London UK

**Keywords:** AVDP ‐ aortic valve disease, electron beam CT/multidetector CT, ICT ‐ imaging, percutaneous intervention, VCOM ‐ vascular complications

## Abstract

**Background:**

Vascular complications after percutaneous transfemoral transcatheter aortic valve implantation (TAVI) are associated with adverse clinical outcomes and remain a significant challenge.

**Aims:**

The purpose of this review is to synthesize the existing evidence regarding the iliofemoral artery features predictive of vascular complications after TAVI on pre‐procedural contrast‐enhanced multidetector computed tomography (MDCT).

**Methods:**

A systematic search was performed in Embase and Medline (Pubmed) databases. Studies of patients undergoing transfemoral TAVI with MDCT were included. Studies with only valve‐in‐valve TAVI, planned surgical intervention and those using fluoroscopic assessment were excluded. Data on study cohort, procedural characteristics and significant predictors of vascular complications were extracted.

**Results:**

We identified 23 original studies involving 8697 patients who underwent TAVI between 2006 and 2020. Of all patients, 8514 (97.9%) underwent percutaneous transfemoral‐TAVI, of which 8068 (94.8%) had contrast‐enhanced MDCT. The incidence of major vascular complications was 6.7 ± 4.1% and minor vascular complications 26.1 ± 7.8%. Significant independent predictors of major and minor complications related to vessel dimensions were common femoral artery depth (>54 mm), sheath‐to‐iliofemoral artery diameter ratio (>0.91–1.19), sheath‐to‐femoral artery diameter ratio (>1.03–1.45) and sheath‐to‐femoral artery area ratio (>1.35). Substantial iliofemoral vessel tortuosity predicted 2–5‐fold higher vascular risk. Significant iliofemoral calcification predicted 2–5‐fold higher risk. The iliac morphology score was the only hybrid scoring system with predictive value.

**Conclusions:**

Independent iliofemoral predictors of access‐site complications in TAVI were related to vessel size, depth, calcification and tortuosity. These should be considered when planning transfemoral TAVI and in the design of future risk prediction models.

**Abbreviations**
CFA: common femoral artery
EIA: external iliac artery
IA: Iliac artery
IFA: Iliofemoral artery
IMS: Iliac morphology score
SEIAR: sheath to external iliac artery ratio
SFAAR: sheath to femoral artery area ratio
SFAR: sheath to femoral artery ratio
SIFAR: sheath to iliofemoral artery ratio
TF: transfemoral

## 1 INTRODUCTION

Transcatheter aortic valve implantation (TAVI) is at least equivalent to surgical aortic valve replacement across the whole spectrum of risk, with numbers expected to grow exponentially in the next 5 years.^1^, ^2^, ^3^ Technological advances have enabled most TAVI procedures to be performed via percutaneous transfemoral (TF) access, using suture‐based vascular closure devices and progressively reduced delivery system sizes. As a result, major vascular complications, as defined by the Valve Academic Research Consortium (VARC), have decreased over time, now in the region of 7%–8%.^4^, ^5^ However, vascular complications after TAVI remain a concern and associated with increased mortality, prolonged hospital admissions and reduced quality of life.^6^, ^7^, ^8^, ^9^ The vast majority of vascular complications in TF‐TAVI occur within the iliofemoral arterial segment.^10^ As we expand into lower risk, younger patients, there is increased focus on the ability to reliably predict and prevent these complications. Meticulous pre‐procedural imaging and iliofemoral vasculature risk assessment are of paramount importance.

Contrast‐enhanced multidetector computed tomography (MDCT) is considered the gold standard for pre‐TAVI assessment.^11^ It offers high spatial resolution and 3‐dimensional assessment of iliofemoral morphology to assist in TF access assessment. Numerous studies have examined the predictive value of iliofemoral vessel size, tortuosity and calcification in determining the risk of periprocedural complications. We, therefore, sought to perform a systematic review of evidence to assimilate all reported iliofemoral predictors of vascular complications (Table 1)^12^ derived from contrast‐enhanced MDCT in patients undergoing percutaneous TF‐TAVI.

**Table 1:** Valve Academic Research Consortium‐3 definition of major and minor access‐related vascular complications (adapted from Généreux et al., 2021).^12^

Major complications	Minor complications
‐ Vascular (arterial or venous) injury (perforation, rupture, dissection, stenosis, ischaemia, arterial or venous thrombosis including pulmonary embolism, arteriovenous fistula, pseudoaneurysm, haematoma, retroperitoneal haematoma, infection) or compartment syndrome resulting in death, VARC type ≥2 bleeding, limb or visceral ischaemia, or irreversible neurologic impairment.	‐ Vascular (arterial or venous) injury (perforation, rupture, dissection, stenosis, ischaemia, arterial or venous thrombosis including pulmonary embolism, arteriovenous fistula, pseudoaneurysm, haematoma, retroperitoneal haematoma, infection) not resulting in death, VARC type ≥2 bleeding, limb or visceral ischaemia, or irreversible neurologic impairment.
‐ Distal embolization (non‐cerebral) from a vascular source resulting in death, amputation, limb or visceral ischaemia, or irreversible end‐organ damage.	‐ Distal embolization treated with embolectomy and/or thrombectomy, not resulting in death, amputation, limb or visceral ischaemia, or irreversible end‐organ damage.
‐ Unplanned endovascular or surgical intervention resulting in death, VARC type ≥2 bleeding, limb or visceral ischaemia, or irreversible neurologic impairment.	‐ Any unplanned endovascular or surgical intervention, ultra‐sound guided compression, or thrombin injection, not resulting in death, VARC type ≥2 bleeding, limb or visceral ischaemia, or irreversible neurologic impairment.
‐ Closure device failure resulting in death, VARC type ≥2 bleeding, limb or visceral ischaemia, or irreversible neurologic impairment.	‐ Closure device failure not resulting in death, VARC type ≥2 bleeding, limb or visceral ischaemia, or irreversible neurologic impairment.

Abbreviation: VARC, Valve Academic Research Consortium.

## 2 METHODS

This study was performed in accordance with the Preferred Reporting Items for Systematic Reviews and Meta‐Analyses (PRISMA) statement (Table S1).^13^ A broad systematic search was performed to identify all relevant studies from Embase and Medline (Pubmed) databases on 26.11.2022 using the following keywords and phrases: (transcatheter aortic valve implantation [All Fields] OR transcatheter aortic valve replacement [All Fields] OR TAVI[All Fields] OR TAVR [All Fields]) AND (access‐site complications [All Fields] OR vascular complications [All Fields] OR access‐related complications [All Fields]). We reviewed the reference lists of included studies to find additional studies. This study did not require an ethical approval.

Two independent reviewers (VA and OC) screened the titles and abstracts of the retrieved citations after removing duplications based on pre‐defined criteria. Potential discrepancies between reviewers were resolved through consensus. The inclusion criteria were (1) any original study published in English language and accompanied by full‐text peer‐reviewed article, (2) evaluating patients undergoing percutaneous TF‐TAVI, (3) reporting anatomical predictors or features associated with vascular or access‐related complications derived from contrast‐enhanced MDCT. Studies investigating risk prediction in valve‐in‐valve TAVI, non‐TF access, planned vascular cut‐down/closure and those using fluoroscopic angiography were excluded, as were case reports and conference abstracts. The full texts of relevant manuscripts were reviewed and data extracted into predefined tables. The quality of eligible studies was evaluated using the Newcastle‐Ottawa assessment scale (0–9 points).^14^

Endpoints of interest were significant predictors of vascular complications identified through univariate tests and independent predictors in multivariable analyses. Reported risk ratios or odds ratios and their corresponding 95% confidence intervals (CI) were extracted when available. Other data extracted included first author, year of publication, TAVI year, country of origin, study design, total number of patients, median age, gender, proportion of patients undergoing TF‐TAVI, proportion of patients with contrast‐enhanced MDCT, percentage of major and minor vascular complications, VARC definition, all examined anatomical predictors, methodology of iliofemoral calcification and tortuosity assessment, brand of TAVI, technique for arterial puncture and range of delivery sheath sizes. Quantitative variables are expressed as mean, standard deviation and percentages.

## 3 RESULTS

### 3.1 Study selection

**Figure 1:**
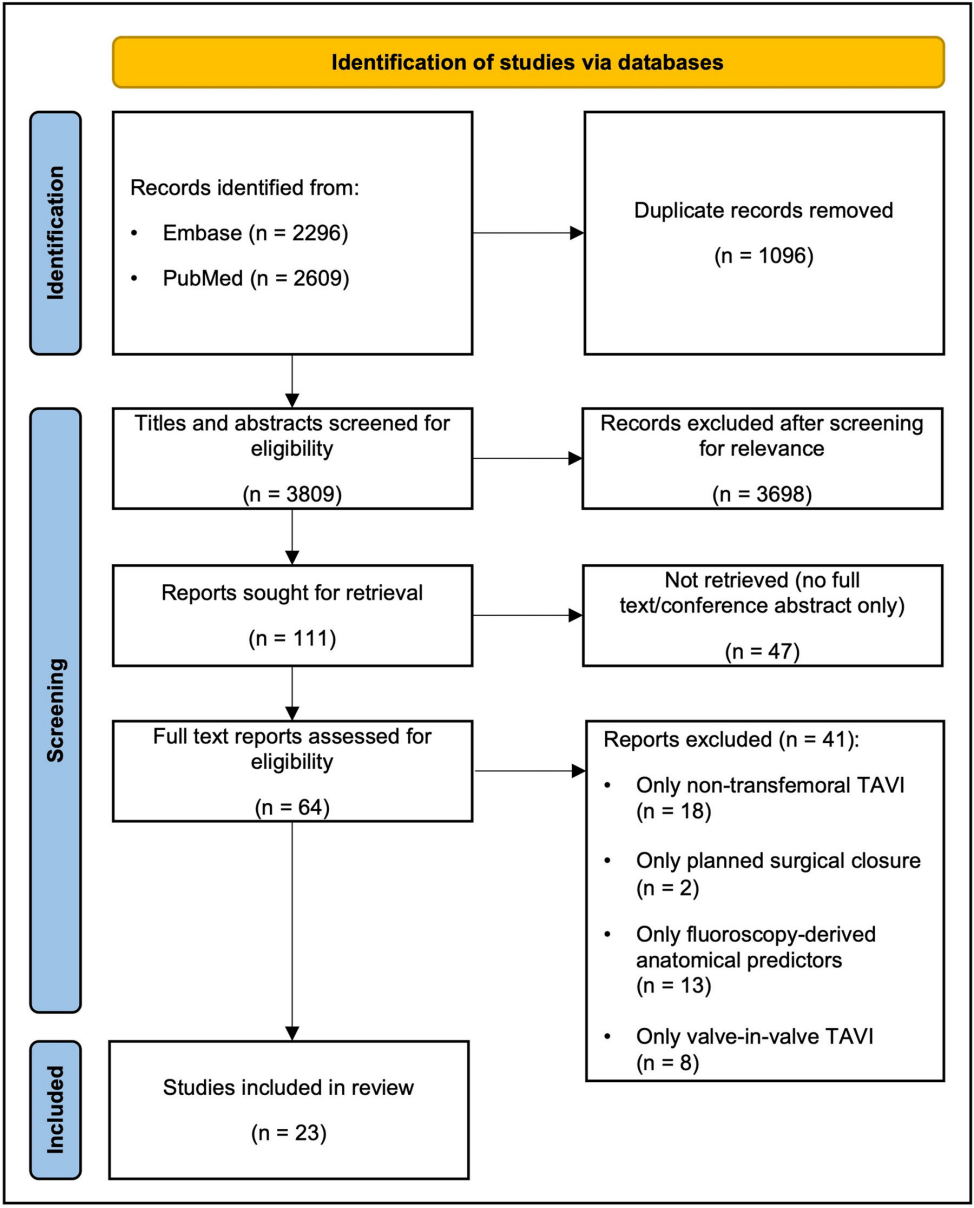
PRISMA flow chart of the included studies. PRISMA, Preferred Reporting Items for Systematic Reviews and Meta‐Analyses; TAVI, transcatheter aortic valve implantation. [Color figure can be viewed at wileyonlinelibrary.com]

Figure 1 shows the PRISMA flow diagram. After removal of 1096 duplicates, a total of 3809 reports were initially identified, of which 3698 were excluded on the basis of screening at the title and abstract level (Figure S1). Of the remaining 111 reports, 64 studies were retrieved in full text and examined for eligibility. Of these, 23 studies involving 8697 patients fulfilled the pre‐specified selection criteria and were deemed eligible for the analysis.

### 3.2 Characteristics of included studies

The key characteristics, design features and predictors of vascular complications of the included studies are summarized in Table 2. All 23 finalized studies were observational, with 18 (78.3%) retrospective cohort studies, 4 (17.4%) prospective cohort studies and 1 (4.3%) case‐control study. The majority of studies were single center (21, 91.3%) with TAVI performed between 2006 and 2020, and were published between 2011 and 2022. Of the included studies, most (22, 95.7%) were of moderate/good quality (Table S2). Most studies (16, 69.6%) were performed in the United States (6, 26.1%) and Europe (Germany: 3, 13.0%; France: 3, 13.0%; Netherlands: 2, 8.7%; and Turkey: 2, 8.7%). The smallest study involved 90 patients and the largest 1497 patients (median: 331, interquartile range: 204). All studies included patients deemed suitable for percutaneous TF‐TAVI following multidisciplinary heart valve team discussion and all procedures were performed using local standard techniques. Therapeutic modification of diseased iliofemoral vessels using balloon angioplasty or intravascular lithotripsy to facilitate TAVI delivery was left to the discretion of the operating physicians. These patients were not explicitly excluded from the analysis in any of the studies. Completely percutaneous TF‐TAVI was performed in 8514 (97.9%) patients, of which 8068 (94.8%) had contrast‐enhanced MDCT. In most studies (22, 95.7%), vascular access site and access‐related complications were categorized using VARC‐2 criteria. Vascular complications were classified as major in 6.7 ± 4.1% patients and minor in 26.1 ± 7.8% patients.

**Table 2:** Characteristics, design features, and key predictors of vascular complications in univariate tests and multivariable analysis of included studies.

Study (Ref. #)	Study year	TAVI year	Region	Design	Patients (*N*)	TF‐TAVI (*N*, %)	MDCT (*N*, %)	Complications (%)	Definition	MDCT‐derived predictors studied	Univariate test predictors (OR/HR, CI, *p* value)	Multivariate predictors (OR/HR, CI, *p* value)
Hayashida et al.^15^	2011	2006–2010	France	Prospective, observational, single center	127	127 (100%)	69 (54%)	Major 17.3% Minor 10.2%	VARC‐1	‐ Minimum IFA diameter.	**Major complications**	**Major complications**
										‐ Degree of calcification.	**SFAR**: *p* = 0.001	**SFAR**: OR 186.2 (4.41–7855.1), *p* = 0.006
										‐ Degree of tortuosity.	**CFA calcification**: *p* = 0.023	**CFA calcification**: OR 3.44 (1.16–10.2), *p* = 0.026
Kadakia et al.^16^	2014	2007–2013	USA	Retrospective, observational, single center	331	211 (63.7%)	321 (97%)	Major 11%	VARC‐2	‐ Minimum IFA diameter.	**All complications**	**All complications**
								Minor 6%			**Minimal IFA diameter < sheath outer diameter**: *p* = 0.02	**Minimal IFA diameter < sheath outer diameter**: OR 1.4 (1.1–1.80, *p* = 0.02 (all)
												**Major Complications**
												**Minimal IFA diameter < sheath outer diameter**: OR 2.0 (1.4–2.9) *p* < 0.001
Krishnaswamy et al.^17^	2014	2006–2012	USA	Retrospective, observational, single center	255	255 (100%)	255 (100%)	Major 4.3%	VARC‐2	‐ Minimum IFA diameter.	**All complications**	**All complications**
								Minor 6.7%		‐ Minimum IFA area.	**CFA MLD**: *p* < 0.001	**SFAR**: OR 8.3 (1.8–39.1), *p* < 0.05
										‐ Degree of calcification.	**CFA MLA**: *p* < 0.001	**SFAAR:** OR 40.1 (2.4–650.0), *p* < 0.05
										‐ Degree of tortuosity.	**EIA MLD**: *p* < 0.006	
											**EIA MLA**: *p* = 0.01	
											**SFAR**: *p* = 0.006	
											**SFAAR**: *p* = 0.008	
Okuyama et al.^18^	2014	2007–2013	USA	Retrospective, observational, single center	386	386 (100%)	283 (73.3%)	Major 13.8%	VARC‐2	‐ Minimum IFA diameter.	**‐**	**Major complications**
										‐ Degree of calcification.		**SIFAR** > **1.12**: OR 32.2 (7.44–139.6), *p* < 0.001
										‐ Degree of tortuosity.		
Reinthaler et al.^19^	2015	2010–2012	Switzerland	Retrospective, observational, single center	132	132 (100%)	103 (78%)	Major 6%	VARC‐2	‐ Minimum IFA diameter/area.	**Major complications**	**Major complications**
								Minor 17%		‐ Degree of tortuosity.	**SIFAR**: OR 64 (1.4–2971), *p* = 0.037	**Circumferential IFA calcification**: OR 5.4,^1^, ^2^, ^3^, ^4^, ^5^, ^6^, ^7^, ^8^, ^9^, ^10^, ^11^, ^12^, ^13^, ^14^, ^15^, ^16^, ^17^, ^18^, ^19^, ^20^, ^21^, ^22^, ^23^, ^24^, ^25^, ^26^, ^27^, ^28^, ^29^, ^30^, ^31^, ^32^, ^33^, ^34^, ^35^, ^36^, ^37^, ^38^, ^39^, ^40^, ^41^ *p* = 0.044
										‐ Degree of calcification.	**Circumferential IFA calcification**: OR 6 (1.2–26), *p* = 0.020	**SIFAR**: OR 280 (0.9–90150), *p* = 0.049
Dencker et al.^25^	2016	2013–2015	Denmark	Retrospective, observational, single center	333	333 (100%)	171 (51%)	Major 4.8%	VARC‐2	‐ Minimum CFA diameter.	**Major complications**	**–**
										‐ Degree of calcification.	**SFAR**: OR 15.44 (2.54–98.4), *p* = 0.004	
											**SFAAR**: OR 9.90 (2.24–43.8), *p* = 0.003	
											**CFA MLD**: OR 0.68 (0.51–0.90), *p* = 0.008	
											**CFA MLA**: OR 0.18 (0.01–0.30), *p* = 0.005	
Uguz et al.^20^	2016	2011–2014	Turkey	Prospective, observational, single center	211	211 (100%)	211 (100%)	Major: 5.7%	VARC‐2	‐ Minimum IFA diameter.	**Major complications**	**Major complications**
								Minor: 10.4%		‐ Degree of calcification.	**IFA MLD**: *p* = 0.000	**IFA calcification**: OR 2.88 (1.14–7.30), *p* = 0.025
										‐ Degree of tortuosity.	**SIFAR**: *p* = 0.000	**SIFAR**: OR 1.91 (1.27–2.87), *p* = 0.001
											**IFA calcification**: *p* = 0.000	
Fonseca et al.^31^	2017	2007–2014	Portugal	Retrospective, observational, single center	140	140 (100%)	138 (98.6%)	Major: 7.1%	VARC‐2	‐ Minimum IFA diameter.	**All complications**	**All complications**
								Minor: 29.3%		‐ IFA calcium score.	**SIFAR**: 17.78 (2.41–130.9), *p* = 0.005	**SIFAR**: HR 14.50 (1.75–120.12), *p* = 0.013
Blakeslee‐Carter et al.^26^	2018	2011–2015	USA	Retrospective, observational, single center	198	198 (100%)	198 (100%)	Major 4%	VARC‐2	‐ Minimum IFA dimeter, area, volume.	**Major complications**	**Major complications**
								Minor 9%			**SFAR**: *p* = 0.001	**IMS**: OR 4 (1.14–14.0), *p* = 0.03
										‐ Degree of calcification.	**SEIAR**: *p* = 0.001	**CFA MLA**: OR 1.25 (1.10–1.58), *p* = 0.039
											**CFA MLD**: *p* = 0.001	
											**CFA MLA**: *p* = 0.001	
											**IMS**: *p* = 0.005	
											**IA calcification**: *p* = 0.001	
van Kesteren et al.^32^	2018	2014–2016	Netherlands	Retrospective, observational, single center	400	400 (100%)	400 (100%)	Major 5.8%	VARC‐2	‐ Minimum IFA diameter.	–	**Major complications**
								Minor 15.0%		‐ Degree of calcification.		**SIFAR**: OR 7.51 (1.61–34.95), *p* = 0.010
										‐ Degree of tortuosity.		
Hammer et al.^29^	2019	2010–2017	Israel	Retrospective, case‐control, propensity matched	90	90 (100%)	90 (100%)		VARC‐2	‐ Minimum IFA volume, dimeter, area.	**All complications**	–
										‐ IFA wall volume.		
										‐ Degree of tortuosity.	**IFA lumen volume**: *p* < 0.001	
										‐ Degree of calcification.		
Urbach et al.^27^	2019	2011–2017	USA	Retrospective, observational, single center	481	481 (100%)	440 (91%)	Major 1.2%	VARC‐2	‐ Minimum CFA and EIA diameter 1 cm below and 2 cm above the IEA, respectively.	**All Complications**	–
								Minor 7.7%			**CFA calcification**: *p* < 0.001	
										‐ Depth of CFA and EIA	**CFA calcification at access site**: *p* = 0.01	
										‐ Position, circumference, and thickness.		
										of calcification.	**Anterior CFA calcification at CFA access site**: *p* = 0.02	
										‐ Tortuosity within each arterial segment.	**Anterior EIA calcification**: *p* = 0.004	
											**CFA MLD**: *p* = 0.04	
											**CFA depth at 45°**: *p* = 0.002	
											**SFAR:** *p* = 0.002	
											**SEIAR**: *p* = 0.012	
Batchelor et al.^23^	2020	2016–2018	USA	Retrospective, observational, single center	303	303 (100%)	303 (100%)	Major 6.3%	VARC‐2	‐ Minimum IFA diameter.	**Major complications**	**Major complications**
											**SFAR** > **0.75**: OR 3.1 (1.2–8.0), *p* = 0.01	
								Minor 9.6%		‐ CFA depth.	**SEIAR**: OR 33 (1.5–794), *p* = 0.02	**Pelvic vessel tortuosity (SFAR** > **0.75):** OR 3.1 (CI: 1.1–9.2), *p* = 0.04
										‐ Degree of CFA calcification.	**CFA MLD**: OR 0.65 (0.43–0.99), *p* = 0.04	
										‐ Pelvic vessel tortuosity.	**EIA MLD**: OR 0.63 (0.42–0.93), *p* = 0.02	
Langouet et al.^21^	2020	2017	France	Prospective, observational, multicentre	479	416 (86.8%)	479 (100%)	Major 2.9%	VARC‐2	‐ Minimum IFA diameter.	**All complications**	**All complications**
								Minor 23.2%		‐ Degree of calcification.	**IFA MLD**: *p* = 0.002	**SIFAR:** OR 6.52 (1.19–21.34), *p* = 0.002
										‐ Degree of tortuosity.	**SIFAR**: *p* < 0.001	**IMS:** OR 1.25 (1.08–1.46), *p* = 0.003
										‐ IMS.		**Moderate‐severe IFA calcification**: OR 2.00 (1.29–3.10), *p* = 0.002
												**Moderate‐severe IFA tortuosity**: OR 2.36 (1.48–3.76), *p* < 0.001
												**Major complications**
												**SIFAR**: OR 31.02 (4.03–238.6), *p* = 0.001
Durand et al.^30^	2021	2013–2018	France	Retrospective, observational, single center	689	689 (100%)	689 (100%)	Major 5.4%	VARC‐2	‐ Minimum IFA diameter.	**Major complications**	**Major complications**
								Minor 9.9%		‐ CFA depth.	**SFAR**: *p* < 0.0001	**SFAR**: HR 8.86 (1.42−55.2), *p* = 0.02
										‐ Degree of calcification.	**Need for Stent Graft**	**Need for Stent Graft**
										‐ Degree of tortuosity.	**CFA depth:** *p* = 0.007	**CFA depth**: HR 1.02 (1.00−1.04), *p* = 0.048
Gonska et al.^36^	2021	2019–2020	Germany	Retrospective, observational, single center	400	400 (100%)	400 (100%)	Major 1.25%	VARC‐2	‐ Minimum CFA diameter.	**All complications**	**All complications**
								Minor 21%		‐ Degree of CFA calcification.	>**20 F sheath**: OR 0.48 (0.30–0.78), *p* = 0.0025	>**20 F sheath:** OR 0.43 (0.25–0.74), *p* = 0.002
Mach et al.^34^	2021	2009–2017	Austria	Retrospective, observational, single center	240	240 (100%)	240 (100%)	Major 2.9%	VARC‐2	‐ Minimum IFA diameter.	**All complications**	**All complications**
								Minor 18.8		‐ CFA depth.	**IFA tortuosity score:** OR 2.44 (1.31–4.54), *p* = 0.005	**IFA tortuosity score**: OR 2.105 (1.09–4.05), *p* = 0.026
										‐ Degree of tortuosity.		
										‐ Degree of calcification.	**Largest single angle**: OR 2.32 (1.11–4.87), *p* = 0.025	
Ruge et al.^33^	2021	2014–2019	Germany	Retrospective, observational, single center	878	878 (100%)	834 (95%)	Major: 9.9%	VARC‐2	‐ CFA diameter.	**All complications**	**All complications**
								Minor: 8.1%		‐ Degree of calcification.	**SFAR**: *p* < 0.001	**SFAR**: OR 1.35 (1.2–1.6), *p* < 0.001
Staudacher et al.^28^	2021	2015–2019	Germany	Retrospective, observational, single center	417	417 (100%)	417 (100%)	Major 8.2%	VARC‐2 and BARC	‐ Minimum IFA diameter.	**All complications**	–
										‐ IFA calcification volume and position (10 cm proximal to the femoral bifurcation.	**CFA MLD 1 cm proximal to CFA bifurcation:** *p* = 0.040	
											**SFAR 1 cm proximal to CFA bifurcation**: *p* = 0.032	
											**Ventral calcification within 5 cm proximal to the CFA bifurcation**: *p* = 0.034	
Cakal et al.^22^	2022	2016–2019	Turkey	Retrospective, observational, single center	223	223 (100%)	223 (100%)	Major 7.6%	VARC‐2	‐ Minimum IFA diameter.	**All complications**	**All complications**
								Minor 11.2%		‐ Degree of calcification.	**Sheath ineligibility (SFAR > md‐SFAR):** *p* = 0.001	**Sheath ineligibility (SFAR > md‐SFAR):** HR 3.7 (1.13–12.53), *p* = 0.031
										‐ Degree of tortuosity.	**IFA MLD**:	
											*p* = 0.035	
											**Sheath ineligibility (guide):** *p* = 0.032	
											**SFAR**: *p* = 0.009	
Honda et al.^24^	2022	2013–2017	Japan	Prospective, observational, multicentre	1497	1497 (100%)	1497 (100%)	Major 11.0%	VARC‐2	‐ Minimum IFA diameter.	**All complications**	**All complications**
								Minor 12.8%			**EIA MLD**: *p* < 0.001	**SFAR**: OR 1.12 (1.03–1.24), *p* = 0.002
										‐ Degree of tortuosity.	**CFA MLD**: *p* < 0.001	
										‐ Degree of calcification.	**SFAR**: *p* < 0.001	
Lux et al.^35^	2022	2019–2020	Netherlands	Retrospective, observational, single center	109	109 (100%)	109 (100%)	Major 9.2%	VARC‐2	‐ Minimum IFA diameter/volume.	**All complications**	**All complications**
								Minor 31.2%		‐ IFA tortuosity index.	**Tortuosity index**: *p* = 0.012	**Angulation** > **49.5° or tortuosity index** > **22.8**: OR 2.72 (1.01–7.33), *p* = 0.048
										‐ IFA calcification.	**Maximal IFA angulation**: *p* = 0.026	**Angulation** >**49.5° and tortuosity index** >**22.8:** OR 5.11 (1.89–13.9), *p* = 0.001
												**Major Complications**
												**Iliofemoral angulation (>49.5°):** OR 7 (1.4–34.8), *p* = 0.017
Miyashita et al.^37^	2022	2018–2020	Finland	Retrospective, observational, single center	378	378 (100%)	378 (100%)	Major 1.9%	VARC‐2	‐ Minimum CFA diameter.	**All complications**	**All complications**
								Minor 4.2%		‐ Degree and position of CFA calcification.	**Anterior CFA calcification (9‐3 o'clock)**: OR 4.74 (1.71–12.1), *p* < 0.002	**Anterior CFA calcification (9‐3 o'clock)**: OR 3.96 (1.32–10.9), *p* = 0.02

*Note*: Values are *n*, *n* (%), or %.

Abbreviations: CFA, common femoral artery; CI, confidence intervals; CIA, common iliac artery; EIA, external iliac artery; HR, hazard ratio; IA, iliac artery; IFA, iliofemoral artery; IMS, iliac morphology score; MDCT, contrast‐enhanced multidetector computed tomography; md‐SFAR, modified SFAR definition; MLD, minimum luminal diameter; OR, odds ratio; SEIAR, sheath to external iliac artery ratio; SFAAR, sheath to femoral artery area ratio; SFAR, sheath to femoral artery ratio; SIFAR, sheath to iliofemoral artery ratio; TF‐TAVI, transfemoral transcatheter aortic valve implantation; VARC, Valve Academic Research Consortium.

Patient and TF‐TAVI device characteristics are summarized in Table S3. The mean age of all patients was 81 ± 2 years and 51% of the patients were female. Self‐expandable valves were implanted in 2298 (26.4%) patients, balloon‐expandable valves in 4122 (47.4%) patients, differential deployment valves in 299 (3.4%) patients and the valve type was not reported in 1978 (22.7%) patients. The size of TAVI delivery sheaths varied from 14F to 24F. Percutaneous arterial puncture was achieved with angiography guidance alone in 7 (30.4%) studies, ultrasound‐guided micro‐puncture alone in 2 (8.7%) studies, angiography or ultrasound in 3 (13.0%) studies and the remaining 11 (47.8%) studies did not comment on the vascular access technique.

### 3.3 Vessel dimensions and depth

A lower minimum lumen diameter of the iliofemoral artery (IFA) (3, 13.4% studies),^20^, ^21^, ^22^ external iliac artery (EIA) (3, 13.0% studies),^17^, ^23^, ^24^ and common femoral artery (CFA) (7, 30.4% studies)^17^, ^23^, ^24^, ^25^, ^26^, ^27^, ^28^ was significantly associated with increased vascular complications (Table 2). A lower minimum lumen area of EIA (1, 4.3% study)^17^ and CFA (3, 13.0% studies)^17^, ^25^, ^26^ was also significantly associated with increased vascular complications. One study reported an association between lower minimum IFA volume and all vascular complications.^29^ Reduced CFA minimum lumen area was an independent risk factor for major vascular complications [odds ratio (OR): 1.25 (CI: 1.10–1.58), *p* = 0.039] in one study with no reported cut‐off.^26^ A greater difference between sheath outer diameter and minimum IFA diameter was an independent predictor of all [OR: 1.4 (CI: 1.1–1.80), *p* = 0.02] and major vascular complications [OR: 2.0 (CI: 1.4–2.9), *p* < 0.001] in one study with no reported threshold.^16^ One study showed that vascular complications were related to greater distance from skin surface to CFA at 45° angle.^27^ One study showed that greater CFA depth was an independent predictor of the need for a stent‐graft after TF‐TAVI [hazard ratio (HR): 1.02 (CI: 1.00–1.04), *p* = 0.048].^30^ The CFA depth that best predicted the need for a stent‐graft was 54 mm (Sensitivity 63.3%, Specificity 40.9%), with area under curve (AUC) of 0.61 suggesting relatively poor predictive accuracy.

### 3.4 Sheath to vessel ratios

Six (26.1%) studies demonstrated higher sheath to iliofemoral artery ratio (SIFAR) to be an independent predictor of access site complications [*All complications*—HR: 14.5 (CI: 1.75–120.12), *p* = 0.013;^31^ OR: 6.52 (CI: 1.19–21.34), *p* = 0.002;^21^ *Major complications*—OR: 280 (CI: 0.9–90150), *p* = 0.049;^19^ OR: 32.2 (CI: 7.44–139.6), *p* < 0.001;^18^ OR: 1.91 (CI: 1.27–2.87), *p* = 0.001;^20^ OR: 7.51 (CI: 1.61–34.95), *p* = 0.010;^32^ OR: 31.02 (CI: 4.03–238.6), *p* = 0.001.^21^] (Table 2). The accuracy of SIFAR thresholds to predict access site complications varied from relatively poor to modest/good. The best reported SIFAR thresholds were >0.92 (AUC: 0.66, Sensitivity: 71.4%, Specificity: 53.4%),^31^ >1.19 (AUC: 0.72, Sensitivity: 91%, Specificity: 67%),^19^ >1.12 (AUC: 0.87, Sensitivity: 94.3%, Specificity: 65.3%;),^18^ >1.11 (AUC: 0.93, Sensitivity: 100%, Specificity: 78.2%),^20^ >1.13 (AUC: 0.63, Sensitivity: 56.6%, Specificity: 62.8%),^32^ and >0.91 (all complications)/>0.95 (major complications) (AUC: 0.62 for major complications, AUC for all complications unknown, Sensitivity and Specificity not reported).^21^ Three studies (13.0%) reported a significant association between higher sheath to external iliac artery ratio (SEIAR) and vascular access‐related complications.^23^, ^26^, ^27^

Increased sheath to femoral artery ratio (SFAR) was significantly associated with access‐related complications in 6 (25.1%) studies.^22^, ^23^, ^25^, ^26^, ^27^, ^28^ A further 5 (21.7%) studies identified SFAR as an independent predictor for access‐site complications in multivariate analysis [*All complications*—OR: 8.3 (CI: 1.8–39.1), *p* < 0.05;^17^ OR: 1.35 (CI: 1.2–1.6), *p* < 0.001;^33^ OR 1.12 (CI: 1.03–1.24), *p* = 0.002;^24^ *Major complications*—OR: 186.2 (CI: 4.41–7855.1), *p* = 0.006;^15^ HR: 8.86 (CI: 1.42–55.2), *p* = 0.02^30^]. SFAR thresholds for predicting vascular complications showed poor‐modest discrimination and were >1.05 (AUC: 0.73, Sensitivity: 66.7%, Specificity: 65.6%),^15^ >1.45 (AUC: 0.68, Sensitivity: 64.2%, Specificity: 67.4%),^17^ and >1.03 (AUC: 0.70, Sensitivity: 67.6%, Specificity: 65.2%).^30^ One study demonstrated that modified SFAR, defined as SFAR greater than or equal to the minimum SFAR recommended in the manufacturer's delivery sheath guidelines, was independently related to all vascular complications (HR: 3.7 (CI: 1.13–12.53), *p* = 0.031), with no reported cut‐off.^22^ Two studies showed that higher sheath to femoral artery area ratio (SFAAR) was associated with vascular complications.^17^, ^25^ One study identified SFAAR as an independent predictor of all vascular complications (OR: 40.1 (CI: 2.4–650.0), *p* < 0.05), with SFAAR >1.35 (AUC: 0.70, Sensitivity: 78.6%, Specificity: 62.9%) providing modest discrimination.^17^

### 3.5 Vessel tortuosity

Fifteen studies (65.2%) evaluated iliofemoral tortuosity for predicting vascular complications (Table S4). Vessel tortuosity was assessed semi‐quantitatively by subjectively grading the severity of tortuosity in 11 (73.3%) studies.^15^, ^17^, ^19^, ^20^, ^21^, ^22^, ^23^, ^24^, ^29^, ^30^, ^32^ Vessel tortuosity was measured objectively using quantitative methods in 4 (26.7%) studies by calculating iliofemoral tortuosity score ([true centreline vessel length/ideal vessel length) − 1) × 100]),^34^, ^35^ maximal degree of angulation,^18^, ^35^ sum of all angles^10^ and degrees of angulation per centimeter of a vessel.^27^

Four studies (26.7%) identified a significant association between the extent of vessel tortuosity and vascular complications.^21^, ^23^, ^34^, ^35^ In multivariate analysis, all four studies demonstrated that iliofemoral tortuosity is an independent risk factor of access‐related complications. Pelvic vessel tortuosity (2 bends ≥90° with SFAR >0.75) resulted in a threefold higher risk of major complications (OR: 3.1 (CI: 1.1–9.2), *p* = 0.04).^23^ Moderate‐severe tortuosity (tortuosity angle 60° to >90°) increased the risk of all complications twofold (OR: 2.36 (CI: 1.48–3.76), *p* < 0.001).^21^ Iliofemoral tortuosity score was identified as an independent predictor of all complications (OR: 2.11 (CI: 1.09–4.05), *p* = 0.026), with a cut‐off >21.2 (AUC: 0.59, Sensitivity 80.8%, Specificity 68.9%) providing poor differentiating ability.^34^ Patients with high maximal iliofemoral angulation (>49.5°) (AUC: unknown, Sensitivity: 57%, Specificity: 70%) or significant tortuosity index (>22.8) (AUC: unknown, Sensitivity: 62%, Specificity: 61%) had twofold increased risk for all access‐related complications in a multivariable model (OR: 2.72 (CI: 1.01–7.33), *p* = 0.048).^35^ The risk increased fivefold in patients with both high angulation and significant tortuosity (OR: 5.11 (CI: 1.89–13.9), *p* = 0.001).^35^ Significant iliofemoral vessel angulation (>49.5°) predicted major complications on its own (OR: 7 (CI: 1.4–34.8), *p* = 0.017).^35^

### 3.6 Vessel calcification

Twenty two (95.6%) studies assessed IFA calcification for predicting TAVI‐related vascular complications (Table S5). Iliofemoral calcification location and severity were graded subjectively using semi‐quantitative methods in 20 (90.9%) studies.^15^, ^17^, ^18^, ^19^, ^20^, ^21^, ^22^, ^23^, ^24^, ^25^, ^26^, ^27^, ^28^, ^29^, ^30^, ^32^, ^33^, ^35^, ^36^, ^37^ Calcification was quantified objectively by applying predefined Hounsfield unit (HU) thresholds in 5 (22.7%) studies^25^, ^28^, ^31^, ^34^, ^35^ and by measuring the maximum circumference and thickness of calcification in 2 (9.1%) studies.^23^, ^27^

Nine (40.9%) studies identified a significant association between iliofemoral calcification and access‐related complications. The calcification severity in the IFA,^20^ iliac artery (IA)^26^ and CFA,^15^, ^27^, ^30^ the presence of circumferential IFA calcification^19^ and anterior calcification of the CFA^27^, ^28^, ^37^ and EIA^27^ have been linked with increased risk. In multivariate analysis, major vascular complications risk was increased threefold by CFA calcification (OR: 3.44 (CI: 1.16–10.2), *p* = 0.026),^15^ fivefold by circumferential IFA calcification (OR: 5.4 (CI: 1–41), *p* = 0.044),^19^ and twofold by moderate‐severe iliofemoral calcification (OR: 2.88 (CI: 1.14–7.30), *p* = 0.025.^20^ All vascular complications increased twofold with moderate‐severe iliofemoral calcification (OR: 2.00 (CI: 1.29–3.10), *p* = 0.002),^21^ and threefold with anterior CFA calcification (OR: 3.96 (CI: 1.32–10.9), *p* = 0.02).^37^

### 3.7 Iliac morphology score

Two (8.7%) studies assessed the iliac morphology score (IMS) for predicting vascular complications.^21^, ^26^ The IMS consists of subjectively assessed IA calcification severity and minimum IA diameter. Each attribute was graded semi‐quantitatively (0–3), with higher scores representing increasingly less favorable morphology. The IMS was a strong independent predictor of major complications (OR: 4 (CI: 1.14–14.0), *p* = 0.03), with score ≥5 achieving good discrimination (AUC: 0.82, Sensitivity: 83%, Specificity: 73%).^26^ However, this finding was not reproducible, with another study showing that IMS can predict all but not major vascular complications (OR: 1.25 (CI: 1.08–1.46), *p* = 0.003) with AUC of 0.58 (Sensitivity, Specificity unknown) suggesting relatively poor discrimination.^17^

## 4 DISCUSSION

**Figure 2:**
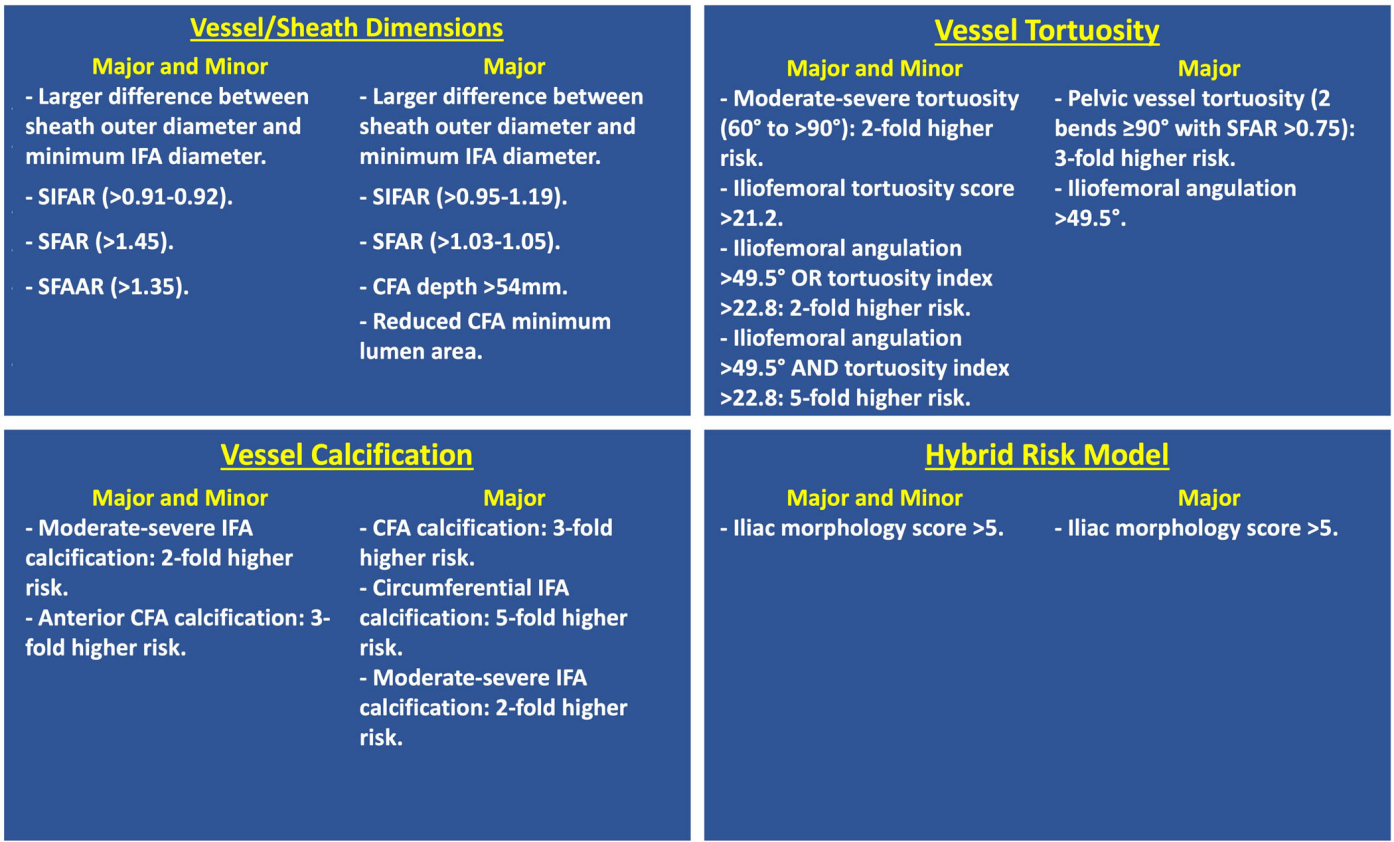
Independent iliofemoral predictors of access site vascular complications in TAVI. CFA, common femoral artery; IFA, iliofemoral artery; SFAAR: sheath to femoral artery area ratio; SFAR, sheath to femoral artery diameter ratio; SIFAR, sheath to iliofemoral artery diameter ratio. [Color figure can be viewed at wileyonlinelibrary.com]

This is the first systematic review to describe all iliofemoral predictors of vascular complications after percutaneous TF‐TAVI from pre‐procedural contrast‐enhanced MDCT. A total of 23 unique studies involving 8697 patients were included, with key independent iliofemoral risk factors summarized in Figure 2.

Percutaneous TF approach is the preferred access strategy for TAVI, with >90% of procedures performed using this route.^38^ However, TF access involves manipulation of large bore sheaths and TAVI delivery systems in the often diseased iliofemoral vasculature, exposing patients to a risk of vascular complications. Despite technological improvements with lower sheath profiles and increased operator experience, the rate of vascular complications remains substantial.^39^ These are associated with increased mortality, poor quality of life, prolonged hospital admissions and increased healthcare costs.^10^ Therefore, there is a clear need for a continuous effort to identify patient and procedural factors associated with increased risk of vascular complications to prevent them during procedure planning.

Contrast‐enhanced MDCT is the gold standard for pre‐procedural iliofemoral vasculature evaluation, TAVI planning and patient selection. MDCT can accurately evaluate iliofemoral vessel dimensions, calcification load and distribution, tortuosity and depth, all of which can assist in selecting the optimal vessel entry site for TAVI.^11^ To this date, there are no specific recommendations on how to summarize a broad range of iliofemoral measurements on MDCT to stratify TAVI recipients into distinct risk categories of vascular complications. Additionally, it is unknown if there is a threshold at which a combination of adverse iliofemoral features should warrant an upfront consideration of alternative non‐TF access. Most of the studies included in this review reported on individual predictors of vascular complications but have not integrated these into a scoring system. The IMS was the only identified semi‐quantitative scoring system for grading the risk of vascular complications in TAVI. However, the strength of this tool has not been consistently demonstrated,^21^, ^26^ with further work required to built on this model.

Most of the included studies investigated the predictors of vascular complications across the whole spectrum of TAVI valves, including older generation devices with larger delivery systems compared with those currently in routine clinical use. The ratio between minimal iliofemoral diameter and sheath outer diameter has been consistently predictive of vascular complications, although with poor/modest predictive accuracy. Furthermore, variable cut‐offs have been reported, making identification of patients at higher risk challenging. This is possibly reflective of TAVI developments over time and transition to smaller sheath sizes with newer generation devices, which are associated with reduced vascular and bleeding complications.^40^, ^41^ However, low‐profile systems have enabled TAVI deliverability to patients with smaller iliofemoral vessels. Therefore, further work is needed to evaluate the relationship between minimum vessel/sheath diameter and vascular complications in the era of new generation TAVI devices.

Some studies have suggested that female gender is a strong predictor of TAVI‐related vascular complications and an important consideration for procedural planning.^17^, ^23^, ^25^, ^33^ Gender‐related differences in iliofemoral morphology on MDCT among TAVI patients remain poorly characterized in the context of other patient‐specific factors. However, the increased risk may be due to the smaller calibre of iliofemoral vessels in females compared to males, resulting is less favorable sheath‐to‐artery ratios.^33^, ^42^

To minimize potential complications associated with significant iliofemoral tortuosity and calcification, accurate and reproducible assessment of these variables is needed. The guidelines recommend describing these factors subjectively and grading into four simple categories, as none, mild, moderate or severe.^11^ This is in keeping with our review, which demonstrates mostly qualitative assessment methods. Qualitative assessment is quick and easy, but the subjective component is liable to inter and intra‐observer variability. It remains to be established if the additional rigour and objectivity of quantitative approaches provide any advantages over and above the subjective methodologies.

Amongst the included studies we demonstrated inconsistency between the severity of iliofemoral tortuosity and vascular complications. This may support wider anecdotal beliefs that even in cases of significant tortuosity, iliofemoral vessels can straighten to allow safe passage of TAVI systems.^11^ However, this approach may further exacerbate the issues of increased sheath manipulation, with additional exertional force which could contribute to vascular complications. Of the available tools, objective assessment of tortuosity with iliofemoral tortuosity score and maximal vessel angulation has demonstrable utility in identifying patients at higher risk of vascular and bleeding complications.^34^, ^35^ Applying these quantitative tools to larger cohorts is needed to validate their utility and to improve our understanding of the role that iliofemoral tortuosity plays in predisposing to vascular complications in TF‐TAVI.

Iliofemoral calcification is an important factor for predicting vascular complications in TF‐TAVI, but this has not been supported consistently across the studies in our review. This heterogeneity may arise because the morphology and protrusion of bulky calcification at specific points within the iliofemoral vessel, such as the puncture site and areas of bifurcation, may be more relevant rather than simply the overall calcification. Further studies are needed to elucidate this by performing detailed segmental iliofemoral plaque analysis.

Overall, some but not all of the studies evaluated in this review found consistent iliofemoral predictors of vascular complications after TAVI. This may be attributed to insufficient power of smaller studies due to the low numbers of vascular complication events. Another confounder that could influence the ability of adverse MDCT‐derived features to predict vascular complications is the technique for percutaneous TF puncture. This was largely unknown and inconsistently reported between studies. Ultrasound‐guided micro‐puncture can localize femoral bifurcation and calcium‐free areas, allowing precise arterial puncture for vascular closure device deployment and TAVI sheath insertion. In a recent meta‐analysis, ultrasound‐guided TF access in TAVI reduced the risk of access‐site vascular and bleeding complications by 50% and ~40%, respectively.^43^ Increased operator experience is another important factor linked with fewer vascular complications and this could be considered alongside anatomical predictors in future models.^44^ Different large‐bore vascular closure methods could have an impact on access‐site complications after TAVI but these were heterogeneous and frequently not reported in the included studies. Vascular closure device failure is not uncommon, occurring in up to 8% of patients,^45^ and linked with adverse iliofemoral characteristics, including small CFA diameter,^46^ SFAR,^47^ and calcification.^48^ This could limit the routine upfront use of “one fits all” closure device strategy, instead warranting pre‐emptive use of specific devices in adverse iliofemoral morphology.

## 5 LIMITATIONS

We reviewed the literature to provide a systematic summary of all available iliofemoral predictors of access‐related complications after TF‐TAVI from contrast‐enhanced MDCT. However, there are several notable limitations. Most selected studies were retrospective and some were relatively small, making them prone to bias regarding valid ascertainment of risk predictors. There was considerable heterogeneity of iliofemoral calcification and tortuosity assessment methodologies, which prohibited performing a comprehensive meta‐analysis. Included studies were published over a wide time frame, which may introduce temporal bias related to technical advances in TAVI and patient care. Some studies included early generation devices using larger delivery sheaths, which are no longer in routine clinical use, that could affect the applicability of predictors derived from these studies to latest generation systems. Predictors of vascular complications related to secondary access site have not been examined in the included studies. This study focussed on identifying key iliofemoral risk predictors and anatomical features associated with other major vascular complications related to aortic dissection and aortic/annular rapture is beyond the scope of this review.

## 6 CONCLUSION

This is the first systematic review to describe all known iliofemoral predictors of vascular complications in percutaneous TF‐TAVI on contrast‐enhanced MDCT. Future studies are needed to devise and validate a simple, objective and reproducible risk score of vascular complications after TF‐TAVI in a contemporary cohort of patients across the spectrum of operative risk. We suggest integrating a combination of quantitative and qualitative measurements to assess iliofemoral dimensions, arterial depth, calcification and tortuosity to assist in the creation of this important systematic decision tool for the Heart Team pre‐procedural TAVI planning.

## CONFLICT OF INTEREST STATEMENT

The authors declare no conflicts of interest.

## Supporting information

Supporting information.

## Data Availability

The data that support the findings of this study are available from the corresponding author upon reasonable request.
