# Supplementary material for: Computed tomography derived anatomical predictors of vascular access complications following transfemoral transcatheter aortic valve implantation: A systematic review
Source: Catheter Cardiovasc Interv. 2023 Nov 22;103(1):169–85. doi: 10.1002/ccd.30918 (PMC10915898; doi:10.1002/ccd.30918)
Supplement: Supplementary file 1 — Supporting information. [file CCD-103-169-s001.docx]

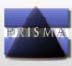
 **Supplementary Table 1.** PRISMA Checklist.

| **Section and Topic** | **Item #** | **Checklist item** | **Location where item is reported** |
| --- | --- | --- | --- |
| **TITLE** | | |  |
| Title | 1 | Identify the report as a systematic review. | 1 |
| **ABSTRACT** | | |  |
| Abstract | 2 | See the PRISMA 2020 for Abstracts checklist. | 2 |
| **INTRODUCTION** | | |  |
| Rationale | 3 | Describe the rationale for the review in the context of existing knowledge. | 4 |
| Objectives | 4 | Provide an explicit statement of the objective(s) or question(s) the review addresses. | 4 |
| **METHODS** | | |  |
| Eligibility criteria | 5 | Specify the inclusion and exclusion criteria for the review and how studies were grouped for the syntheses. | 5 |
| Information sources | 6 | Specify all databases, registers, websites, organisations, reference lists and other sources searched or consulted to identify studies. Specify the date when each source was last searched or consulted. | 5 |
| Search strategy | 7 | Present the full search strategies for all databases, registers and websites, including any filters and limits used. | 5 |
| Selection process | 8 | Specify the methods used to decide whether a study met the inclusion criteria of the review, including how many reviewers screened each record and each report retrieved, whether they worked independently, and if applicable, details of automation tools used in the process. | 5 |
| Data collection process | 9 | Specify the methods used to collect data from reports, including how many reviewers collected data from each report, whether they worked independently, any processes for obtaining or confirming data from study investigators, and if applicable, details of automation tools used in the process. | 5 |
| Data items | 10a | List and define all outcomes for which data were sought. Specify whether all results that were compatible with each outcome domain in each study were sought (e.g. for all measures, time points, analyses), and if not, the methods used to decide which results to collect. | 6 |
|  | 10b | List and define all other variables for which data were sought (e.g. participant and intervention characteristics, funding sources). Describe any assumptions made about any missing or unclear information. | 6 |
| Study risk of bias assessment | 11 | Specify the methods used to assess risk of bias in the included studies, including details of the tool(s) used, how many reviewers assessed each study and whether they worked independently, and if applicable, details of automation tools used in the process. | 5 |
| Effect measures | 12 | Specify for each outcome the effect measure(s) (e.g. risk ratio, mean difference) used in the synthesis or presentation of results. | 6 |
| Synthesis methods | 13a | Describe the processes used to decide which studies were eligible for each synthesis (e.g. tabulating the study intervention characteristics and comparing against the planned groups for each synthesis (item #5)). | 5 |
|  | 13b | Describe any methods required to prepare the data for presentation or synthesis, such as handling of missing summary statistics, or data conversions. | 5 |
|  | 13c | Describe any methods used to tabulate or visually display results of individual studies and syntheses. | 5 |
|  | 13d | Describe any methods used to synthesize results and provide a rationale for the choice(s). If meta-analysis was performed, describe the model(s), method(s) to identify the presence and extent of statistical heterogeneity, and software package(s) used. | N/A |
|  | 13e | Describe any methods used to explore possible causes of heterogeneity among study results (e.g. subgroup analysis, meta-regression). | N/A |
|  | 13f | Describe any sensitivity analyses conducted to assess robustness of the synthesized results. | N/A |
| Reporting bias assessment | 14 | Describe any methods used to assess risk of bias due to missing results in a synthesis (arising from reporting biases). | N/A |
| Certainty assessment | 15 | Describe any methods used to assess certainty (or confidence) in the body of evidence for an outcome. | N/A |
| **RESULTS** | | |  |
| Study selection | 16a | Describe the results of the search and selection process, from the number of records identified in the search to the number of studies included in the review, ideally using a flow diagram. | 6 |
|  | 16b | Cite studies that might appear to meet the inclusion criteria, but which were excluded, and explain why they were excluded. | N/A |
| Study characteristics | 17 | Cite each included study and present its characteristics. | Tables 2 |
| Risk of bias in studies | 18 | Present assessments of risk of bias for each included study. | Supplemental Table 2 |
| Results of individual studies | 19 | For all outcomes, present, for each study: (a) summary statistics for each group (where appropriate) and (b) an effect estimate and its precision (e.g. confidence/credible interval), ideally using structured tables or plots. | Tables 2,3,4 |
| Results of syntheses | 20a | For each synthesis, briefly summarise the characteristics and risk of bias among contributing studies. | 6 |
|  | 20b | Present results of all statistical syntheses conducted. If meta-analysis was done, present for each the summary estimate and its precision (e.g. confidence/credible interval) and measures of statistical heterogeneity. If comparing groups, describe the direction of the effect. | N/A |
|  | 20c | Present results of all investigations of possible causes of heterogeneity among study results. | N/A |
|  | 20d | Present results of all sensitivity analyses conducted to assess the robustness of the synthesized results. | N/A |
| Reporting biases | 21 | Present assessments of risk of bias due to missing results (arising from reporting biases) for each synthesis assessed. | N/A |
| Certainty of evidence | 22 | Present assessments of certainty (or confidence) in the body of evidence for each outcome assessed. | N/A |
| **DISCUSSION** | | |  |
| Discussion | 23a | Provide a general interpretation of the results in the context of other evidence. | 12 |
|  | 23b | Discuss any limitations of the evidence included in the review. | 16 |
|  | 23c | Discuss any limitations of the review processes used. | 16 |
|  | 23d | Discuss implications of the results for practice, policy, and future research. | 16 |
| **OTHER INFORMATION** | | |  |
| Registration and protocol | 24a | Provide registration information for the review, including register name and registration number, or state that the review was not registered. | N/A |
|  | 24b | Indicate where the review protocol can be accessed, or state that a protocol was not prepared. | N/A |
|  | 24c | Describe and explain any amendments to information provided at registration or in the protocol. | N/A |
| Support | 25 | Describe sources of financial or non-financial support for the review, and the role of the funders or sponsors in the review. | 18 |
| Competing interests | 26 | Declare any competing interests of review authors. | 18 |
| Availability of data, code and other materials | 27 | Report which of the following are publicly available and where they can be found: template data collection forms; data extracted from included studies; data used for all analyses; analytic code; any other materials used in the review. | N/A |


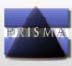


*From:*  Page MJ, McKenzie JE, Bossuyt PM, Boutron I, Hoffmann TC, Mulrow CD, et al. The PRISMA 2020 statement: an updated guideline for reporting systematic reviews. BMJ 2021;372:n71. doi: 10.1136/bmj.n71

For more information, visit: <http://www.prisma-statement.org/>

**Supplementary Table 2.** The Newcastle-Ottawa Assessment Scale for studies

| Studies | Exposed cohort representative? | Selection of non-exposed cohort? | Ascertainment of exposure? | Outcome at baseline? | Controls for important factors? | Controls for other confounders? | Assessments of outcome? | Adequacy of follow-up duration? | Adequacy of lost to follow up? | **Quality score** |
| --- | --- | --- | --- | --- | --- | --- | --- | --- | --- | --- |
| Hayashida *et al. (2011)* | * | N/A | * | * | * | * |  | * | * | 7 |
| Kadakia *et al. (2014)* | * | N/A | * | * |  |  | * | * | * | 6 |
| Krishnaswamy *et al. (2014)* | * | N/A | * | * | * | * | * | * | * | 8 |
| Okuyama *et al. (2014)* | * | N/A | * | * | * |  | * | * | * | 7 |
| Reinthaler *et al. (2015)* | * | N/A | * | * | * |  | * | * | * | 7 |
| Dencker *et al. (2016)* | * | N/A | * | * | * |  | * | * | * | 7 |
| Uguz *et al. (2016)* | * | N/A | * | * | * | * | * | * | * | 8 |
| Fonseca *et al. (2017)* | * | N/A | * | * | * |  | * | * | * | 7 |
| Blakeslee-Carter *et al. (2018)* | * | N/A | * | * | * |  | * | * | * | 7 |
| van Kesteren *et al. (2018)* | * | N/A | * | * | * | * | * | * | * | 8 |
| Hammer *et al. (2019)* |  | N/A | * |  | * |  |  | * | * | 4 |
| Urbach *et al. (2019)* | * | N/A | * | * |  |  | * | * | * | 6 |
| Batchelor *et al. (2020)* | * | N/A | * | * | * | * | * | * | * | 8 |
| Langouet *et al. (2020)* | * | N/A | * | * | * | * | * | * | * | 8 |
| Durand *et al. (2021)* | * | N/A | * | * | * |  | * | * | * | 7 |
| Gonska *et al. (2021)* | * | N/A | * | * | * |  | * | * | * | 7 |
| Mach *et al. (2021)* | * | N/A | * | * | * | * | * | * | * | 8 |
| Ruge *et al. (2021)* | * | N/A | * | * | * |  | * | * | * | 7 |
| Staudacher *et al. (2021)* | * | N/A | * | * |  |  | * | * | * | 6 |
| Cakal *et al. (2022)* | * | N/A | * | * | * |  | * | * | * | 7 |
| Honda *et al. (2022)* | * | N/A | * | * |  | * | * | * | * | 7 |
| Lux *et al. (2022)* | * | N/A | * | * | * | * | * | * | * | 8 |
| Miyashita *et al. (2022)* | * | N/A | * | * | * |  | * | * | * | 7 |

The methodological quality of observational studies was performed using Newcastle–Ottawa scale quality scale. Each asterisk in the Newcastle-Ottawa Scaling System represents responses of the biases questionnaire. Each bias assessment part gets one star except comparability that gets a maximum of 2 stars. Each star counts towards the total score. Score <5 represents poor quality, 5-6 represents moderate quality and 7 to 9 are considered as good quality.

(N/A: not applicable – all included studies evaluated patients that had to undergo transcatheter aortic valve implantation to be included in the analysis.)

**Supplementary Table 3.** Patient and transfemoral TAVI implantation characteristics of included studies.

| **Study (Ref. #)** | **Age (N)** | **Male (%)** | **Valve type (N, %)** | **US/Angiography-guided puncture (%)** | **Sheath Size** |
| --- | --- | --- | --- | --- | --- |
| Hayashida *et al.* 2011 *(33)* | 83 | 49% | CoreValve: 27 (21.3%)  Sapien/XT: 100 (78.7%) | Angiography (100%) | 18F – 24F |
| Kadakia *et al.* 2014 *(26)* | 85 | 48% | Sapien XT: 49 (14.8%)  Sapien: 282 (85.2%) | Mostly angiography | 18F – 24F |
| Krishnaswamy *et al.* 2014 *(18)* | 81 | 58% | Sapien/Sapien XT: 255 (100%) | Unavailable | 18F – 24F |
| Okuyama *et al.* 2014 *(30)* | 84 | 55% | Sapien/XT: 386 (100%) | Unavailable | 16F – 24F |
| Reinthaler *et al.* 2015 *(29)* | 82 | 53% | CoreValve: 40 (30%)  Sapien XT: 92 (70%) | Angiography (78%) | 18F – 20F |
| Dencker *et al.* 2016 *(21)* | 80 | 52% | Sapien 3: 37 (11%)  CoreValve: 222 (67%)  Portico: 31 (9%)  Evolut R: 16 (5%)  LOTUS: 27 (8%) | Angiography (100%) | Unavailable |
| Uguz *et al.* 2016 *(15)* | 78 | 39% | Sapien XT: 147 (70%)  CoreValve: 64 (30%) | Unavailable | 16F – 20F |
| Fonseca *et al.* 2017 *(28)* | 78 | 48% | CoreValve: 91 (65%)  Evolut R: 3 (2.1%)  Sapien XT: 29 (20.7%)  Sapien 3: 17 (12.1%) | Angiography (100%) | 14F – 18F |
| Blakeslee-Carter *et al.* 2018 *(22)* | 81 | 51% | Sapien XT: 80 (40.4%)  Sapien: 59 (29.8%)  Sapien S3: 6 (2.5%)  CoreValve: 50 (25.3%)  DirectFlow: 3 (1.5%) | Unavailable | 14F – 24F |
| van Kesteren *et al.* 2018 *(31)* | 82 | 46% | Sapien 3: 400 (100%) | Angiography (100%) | 14F – 16F |
| Hammer *et al.* 2019 *(25)* | 83 | 40% | Corevalve: 25 (27.8%)  Evolut R: 36 (40%)  Sapien XT: 4 (4.4%)  Sapien 3: 19 (21.1%)  LOTUS: 6 (6.7%) | Unavailable | 14F – 20F |
| Urbach *et al.* 2019 *(23)* | 82 | 63% | Unavailable: 481 (100%) | Unavailable | Unavailable |
| Batchelor *et al.* 2020 *(19)* | 80 | 51% | Edwards: 136 (45%)  Medtronic: 163 (54%)  Other: 4 (1.3%) | Ultrasound (100%) | 14F – 16F |
| Langouet *et al.* 2020 *(16)* | 83 | 54% | Evolut R: 187 (39%)  Sapien 3: 289 (60%)  Portico: 3 (1%) | Unavailable | 14F – 16F |
| Durand *et al.* 2021 *(27)* | 84 | 46% | Corevalve, Evolut R/PRO: 77 (12%)  Sapien XT/3: 612 (88%) | Angiography (100%) | 14F – 20F |
| Gonska *et al.* 2021 *(36)* | 80 | 46% | Evolut R/Pro: 200 (50%)  LOTUS: 200 (50%) | Angiography (100%) | 16F – 20F |
| Mach *et al.* 2021 *(34)* | 83 | 37% | Balloon expanding valve: 69 (28.8%)  Self-expanding valve: 171 (71.2%) | Unavailable | Unavailable |
| Ruge *et al.* 2021 *(32)* | 80 | 52% | Evolut R: 418 (47.6%)  Evolut PRO: 41 (4.7%)  Sapien 3: 405 (46%)  Sapien Ultra: 14 (1.6%) | Angiography (100%) | 14F – 20F |
| Staudacher *et al.* 2021 *(24)* | 81 | 46% | Edwards: 282 (67.6%)  Medtronic: 83 (19.9%)  Directflow: 45 (11.0%)  Symetis: 6 (1.4%)  Jenavalve: 1 (2.4%) | Unavailable | Unavailable |
| Cakal *et al.* 2022 *(17)* | 79 | 49% | Sapien XT: 113 (50.7%)  Sapien 3: 30 (13.5%)  Evolut R: 60 (26.9%)  Portico: 20 (8.9%) | Unavailable | 14F – 20F |
| Honda *et al.* 2022 *(20)* | 85 | 26% | Corevalve, Evolut R, Sapien XT/3: 1497 (100%) | Unavailable | 14F – 20F |
| Lux *et al.* 2022 *(35)* | 79 | 55% | Edwards: 65 (60%)  Medtronic: 44 (40%) | Ultrasound (23%) | 14F – 20F |
| Miyashita *et al.* 2022 *(37)* | 81 | 53% | Sapien 3/Ultra: 145 (38.3%)  ACURATE neo: 110 (29.1%)  Evolut R/Pro: 91 (24.1%)  Allegra: 9 (2.4%)  Portico: 9 (2.4%)  LOTUS: 14 (3.7%) | Ultrasound (100%) | 14F – 18F |

**Supplementary Table 4.** Summary of studies and iliofemoral tortuosity assessment methods for vascular complications risk prediction in TF-TAVI, stratified by those with and without significant findings in univariate tests or multivariable analyses.

| **Study (Ref. #)** | **Description of tortuosity assessment** | **Methodology** | **Univariate test predictors**  **(OR/HR, CI, p value)** | **Multivariate predictors**  **(OR/HR, CI, p value)** |
| --- | --- | --- | --- | --- |
| **Studies with significant findings** | | | | |
| Batchelor *et al.* 2020 *(19)* | - Pelvic vessel tortuosity was defined as at least 2 bends ≥90°. | Semi-quantitative | **All Complications**  **Pelvic vessel tortuosity:** p=0.69 | **Major Complications**  **Pelvic vessel tortuosity (SFAR>0.75)**: OR 3.1 (CI 1.1-9.2), p=0.04 |
| Langouet *et al.* 2020 *(16)* | - IFA tortuosity was graded as none (tortuosity angle <30° at any point of iliofemoral access), mild (tortuosity angle ranging from 30°-60°), moderate (tortuosity angle ranging from 60°-90°), severe (tortuosity angle >90°). | Semi-quantitative | - | **All Complications**  **Moderate-severe tortuosity**: OR 2.36 (1.48-3.76), p<0.001 |
| Mach *et al.* 2021 *(34)* | - IFA tortuosity score [((true vessel length/ideal vessel length)-1)*100], largest single angle (LSA), Sum of all measured angles. | Quantitative | **All Complications**  **IFA tortuosity score**: OR 2.44 (1.31-4.54), p=0.005  **LSA**: OR 2.32 (1.11-4.87), p=0.025 | **All Complications**  **IFA tortuosity score:** OR 2.11 (1.09-4.05), p=0.026 |
| Lux *et al.* 2022 *(35)* | - IFA tortuosity score [((true vessel length/ideal vessel length)-1)*100] and maximal angulation over the vessel centreline utilising a dedicated tool with arm lengths of 15 mm. | Quantitative | **All Complications**  **Tortuosity index:** p=0.012  **Maximum angulation:** p=0.026 | **All Complications**  **Angulation >49.5° or tortuosity index >22.8**: OR 2.72 (1.01-7.33), p=0.048  **Angulation >49.5° and tortuosity index >22.8:** OR 5.11 (1.89-13.9), p=0.001  **Major Complications**  **Iliofemoral angulation (>49.5°):** OR 7 (1.4-34.8), p=0.017 |
| **Studies with no significant findings** | | | | |
| Hayashida *et al.* 2011 *(33)* | - Tortuosity was graded semi-quantitively as 0 (none), 1 (mild, 30°-60°), 2 (moderate, 60°-90°) and 3 (severe, ≥90°). | Semi-quantitative | **Major Complications**  **CFA tortuosity (0-3):** p=0.709  **IA tortuosity (0-3):** p=0.459 | **-** |
| Krishnaswamy *et al.* 2014 *(18)* | - Maximum IFA tortuosity graded as <45°, 45–90° or >90°. | Semi-quantitative | **All Complications**  **Maximum degree of tortuosity:** p=0.55 | **-** |
| Okuyama *et al.* 2014 *(30)* | - Maximum IFA tortuosity angle, measured between 2 equidistant points that generate arms extending along the centre-line of the vessel in opposite directions. | Quantitative | **All Complications**  **Maximum tortuosity angle:** p>0.05 | **-** |
| Reinthaler *et al.* 2015 *(29)* | - Tortuosity was graded semi-quantitively as 0 (none), 1 (mild, 30°-60°), 2 (moderate, 60°-90°) and 3 (severe, ≥90°). | Semi-quantitative | **Major Complications**  **Tortuosity (0-3):** OR 0.6 (0.2-1.7), p=0.311 | **Major Complications**  **Tortuosity (0-3):** OR 0.9 (0.2-4.6), p=0.941 |
| Uguz *et al.* 2016 *(15)* | - IFA tortuosity score graded semi-quantitively (0-3). | Semi-quantitative | **Major Complications**  **Tortuosity (0-3):** p=0.289 | - |
| van Kesteren *et al.* 2018 *(31)* | - IFA tortuosity was graded as none, mild, moderate, severe. | Semi-quantitative | **Major Complications**  **Moderate-severe tortuosity:** OR 1.27 (0.42–3.89), p=0.68 | **-** |
| Hammer *et al.* 2019 *(25)* | - IFA tortuosity scored semi-quantitatively as 0 (none), 1 (mild, 30°-60°), 2 (moderate, 60°-90°) and 3 (severe, >90°). | Semi-quantitative | **All Complications**  **Tortuosity (0-3):** p=0.609 | **-** |
| Urbach *et al.* 2019 *(23)* | - Tortuosity of CFA and EIA measured using a dedicated tool (°/cm). | Quantitative | **All Complications**  **CFA tortuosity (°/cm)**: p=0.390  **EIA tortuosity (°/cm)**: p=0.870 | **-** |
| Durand *et al.* 2021 *(27)* | - IFA axis tortuosity was assessed using a semi-quantitative score as 0 (none), 1 (mild, 30°-60°), 2 (moderate, 60°-90°), 3 (severe, >90°). | Semi-quantitative | - | **Major Complications**  **Tortuosity (0-3):** HR 0.64 (0.32−1.28), p=0.21  **Need for stent-graft**  **Tortuosity (0-3):** HR 1.01 (0.60−1.71), p=0.97 |
| Cakal *et al.* 2022 *(17)* | - IFA tortuosity was semi-quantitively graded as none, mild (30°-60°), moderate (60°-90°), and severe (90°). | Semi-quantitative | **All Complications**  **Tortuosity ≥ moderate**: p=0.986 | - |
| Honda *et al.* 2022 *(20)* | - Access route tortuosity scored semi-quantitively (0-3). | Semi-quantitative | **All Complications**  **Tortuosity (0-3)**: p=0.61 | **-** |

(CFA: common femoral artery, CI: confidence intervals, HR: hazard ratio, IA: iliac artery, IFA: iliofemoral artery, LSA: largest single angle, OR: odds ratio, SFAR: sheath to femoral artery ratio.)

**Supplementary Table 5.** Summary of studies and iliofemoral calcification assessment methods for vascular complications risk prediction in TF-TAVI, stratified by those with and without significant findings in univariate tests or multivariable analyses.

| **Study (Ref. #)** | **Description of calcification assessment** | **Methodology** | **Univariate test predictors**  **(OR/HR, CI, p value)** | **Multivariate predictors**  **(OR/HR, CI, p value)** |
| --- | --- | --- | --- | --- |
| **Studies with significant findings** | | | | |
| Hayashida *et al.* 2011 *(33)* | - Calcification of the IFA vessels (CFA, IA) was graded as 0 (none), 1 (mild), 2 (moderate) and 3 (severe). | Semi-quantitative | **Major Complications**  **CFA calcification (0-3)**: p=0.023 | **Major Complications**  **CFA calcification (0-3)**: OR 3.44 (1.16-10.2), p=0.026 |
| Reinthaler *et al.* 2015 *(29)* | - IFA calcification was graded as 0 (none), 1 (mild), 2 (moderate) and 3 (severe).  - Presence of circumferential IF calcification. | Semi-quantitative | **Major Complications**  **Circumferential IFA calcification**: OR 6 (1.2-26), p=0.020 | **Major Complications**  **Circumferential IFA calcification**: OR 5.4 (1-41), p=0.044 |
| Uguz *et al.* 2016 *(15)* | - IFA calcification was scored 0-3. | Semi-quantitative | **Major Complications**  **IFA calcification (0-3):** p=0.000 | **Major Complications**  **IFA calcification (0-3):** OR 2.88 (1.14-7.30), p=0.025 |
| Blakeslee-Carter *et al.* 2018 *(22)* | - CFA and IA calcification was graded as 0 (none), 1 (mild, covering < 25% of the vessel length), 2 (moderate, covering 25%-50% of the vessel length), 3 (severe, covering > 50% of the vessel length/circumferential calcification at any point). | Semi-quantitative | **Major Complications**  **IA calcification (0-3)**: p=0.001  **IMS:** p=0.005 | **Major Complications**  **IMS:** OR 4 (1.14-14.0), p=0.03 |
| Urbach *et al.* 2019 *(23)* | - Presence, position, circumference (in degrees) and thickness of calcification at the sites for CFA and EIA.  - The sites of CFA and EIA measurements were defined as 1 cm below and 2 cm above the inferior epigastric artery, respectively. | Semi-quantitative  Quantitative | **All Complications**  **Calcification anywhere in CFA**: p<0.001  **CFA calcification at access site**: p=0.01  **Anterior calcification at CFA**: p=0.02  **Anterior calcification of EIA**: p=0.004 | - |
| Langouet *et al.* 2020 *(16)* | - Calcification of IF access was graded as 0 (none), 1 (mild, calcification <25% of the vessel length), 2 (moderate, calcification ranging from 25% to 50%) and 3 (severe, calcification >50% or circumferential). | Semi-quantitative | - | **All Complications**  **Moderate-severe IFA calcification:** OR 2.00 (1.29-3.10), p=0.002  **IMS:** OR 1.25 (1.08-1.46), p=0.003 |
| Durand *et al.* 2021 *(27)* | - Calcification at the puncture site were quantified as: 0 (none), 1 (<30% of the vessel circumference), 2 (30% - 50% of the vessel circumference, 3 (>50% of the vessel circumference), 4 (100% of the vessel circumference or totality of anterior wall). | Semi-quantitative | **Major Complications**  **Puncture site (CFA) calcification (0-3):** p=0.02 | **Major Complications**  **Puncture site (CFA) calcification (0-3):** HR 1.00 (0.58−1.73), p=0.99  **Need for Stent Graft**  **Puncture site (CFA) calcification (0-3):** HR 0.81 (0.50-1.32), p=0.40 |
| Staudacher *et al.* 2021 *(24)* | - Calcification volume (mm^3^) within vascular access site 10 proximal to CFA bifurcation was measured on contract-enhanced MDCT scans using the threshold of 600 HU.  - Presence of ventral calcification recorded. | Semi-quantitative  Quantitative | **Major Complications**  **Anterior calcification within the first 5 cm proximal to the CFA bifurcation**: p=0.034 | **-** |
| Miyashita *et al.* 2022 *(37)* | - Presence of anterior CFA calcification (9-o’clock to 3-o’clock position) noted.  - CFA calcification was graded as 0 (none), 1 (mild), 2 (moderate) and 3 (severe). | Semi-quantitative | **All Complications**  **Anterior CFA calcification (9-3 o’clock)**: OR 4.74 (1.71-12.1), p<0.002 | **All Complications**  **Anterior CFA calcification (9-3 o’clock)**: OR 3.96 (1.32-10.9), p=0.02 |
| **Studies with no significant findings** | | | | |
| Krishnaswamy *et al.* 2014 *(18)* | - Degree of calcification at the level of the CIA, EIA and CFA was graded as 1 (total circumferential arc <90°), 2 (90–180°), 3 (180–270°) and 4 (>270°). | Semi-quantitative | **All Complications**  **Mean calcification score:** CFA (p=0.4), EIA (p=0.3), CIA (p=0.72) | - |
| Okuyama *et al.* 2014 *(30)* | - IFA calcification was graded as 0 (none), 1 (mild), 2 (moderate) and 3 (severe). | Semi-quantitative | **All Complications**  **IFA calcification (moderate-severe)** p=0.18 | **-** |
| Dencker *et al.* 2016 *(21)* | - CFA calcification (puncture site) was graded as none, 1 (mild, <25% of the circumference), 2 (moderate, 25%-50% of the circumference), 3 (severe, >50% of the circumference).  - Calcification volume (mm^3^) was measured at the puncture site including a 10-mm vessel segment proximal to the puncture with an optimised preset window width/level of HU. | Semi-quantitative  Quantitative | **All Complications**  **CFA calcification (0-3):** OR 0.93 (0.44-1.96), p=0.855  **Puncture site calcium volume-anterior:** OR 1.00 (0.99-1.02), p=0.873  **CFA calcification (0-3):** OR 1.11 (0.75-1.64), p=0.597  **Puncture site calcium volume-posterior:** OR 1.00 (0.99-1.00), p=0.550  **CFA calcification (0-3):** OR 1.09 (0.71-1.68), p=0.698  **Puncture site calcium volume-total:** OR 1.00 (0.99-1.00), p=0.498 | **-** |
| Fonseca *et al.* 2017 *(28)* | - The calcium score of the IF access was obtained from contrast-enhanced MDCT images using semi-automated software and different calcium thresholds according to mean luminal attenuation (LA) at aortic root. Calcium thresholds were defined as 600 HU for LA <500 HU, 700 HU for LA 500-600 HU and 800 HU for LA >600 HU. | Quantitative | **All Complications**  **IFA calcium score**: HR 1.00 (1.00-1.00), p=0.097 | **All Complications**  **IFA calcium score**: HR 1.00 (1.00-1.00), p=0.101 |
| van Kesteren *et al.* 2018 *(31)* | - IFA calcification was graded as none, mild, moderate or severe. | Semi-quantitative | **Major Complications**  **Moderate-severe IFA calcification:** OR 2.35 (0.98-5.65), p=0.056 | **-** |
| Hammer *et al.* 2019 *(25)* | - IFA calcification scored as 0 (none), 1 (mild), 2 (moderate) and 3 (severe). | Semi-quantitative | **All Complications**  **IFA calcification (0-3):** p=0.571 | **-** |
| Batchelor *et al.* 2020 *(19)* | - Maximum arc of circumferential CFA calcification (in degrees) on the non-contrast MDCT axial images.  - Location of CFA calcification (anterior, medial, lateral or posterior). | Semi-quantitative  Quantitative | **Major Complications**  **Maximum CFA calcification arc:** OR 0.24 (0.03–1.9) 0.17, p=0.17  **Anterior CFA calcification**: OR 2.5 (0.27–23), p=0.42 | **-** |
| Gonska *et al.* 2021 *(36)* | - CFA calcification classified as none, mild (calcification of <25% of the circumference and not relevant protrusion into the lumen) or severe (calcification of >25% of the circumference, more than two spots, or relevant protrusion into the lumen). | Semi-quantitative | **All Complications**  **CFA calcification (0-3):** OR 0.82 (0.49–1.35), p=0.42 | **-** |
| Mach *et al.* 2021 *(34)* | - IFA calcification burden (mm^3^) on contrast-enhanced MDCT scans using the threshold of 130 HU. | Quantitative | **All Complications**  **IFA calcification load:** OR 1 (1-1), p=0.784 | - |
| Ruge *et al.* 2021 *(32)* | - Vessel calcification at access site (between femoral bifurcation and cranial margin of the hip) graded as none, minimal, moderate or severe based on qualitative, visual assessment.  - Calcification grade determined for anterior, posterior, lateral and medial vessel walls separately. | Semi-quantitative | - | **All Complications**  **Access site moderate-severe calcification:** OR 0.93 (0.61-1.40), p=0.7 |
| Cakal *et al.* 2022 *(17)* | - IFA calcification was graded as none, mild (90° of total circumferential arc), moderate (90°–180° of total circumferential arc), marked (180°–270° of total circumferential arc) and severe calcification (>270° of total circumferential arc). | Semi-quantitative | **All Complications**  **IFA calcification ≥ moderate**: p=0.219 | - |
| Honda *et al.* 2022 *(20)* | - Access route calcification scored 0-3. | Semi-quantitative | **All Complications**  **Access site calcium score (0-3):** p=0.2 | **-** |
| Lux *et al.* 2022 *(35)* | - Calcification volume (mm^3^) was measured between the aortic bifurcation and the femoral bifurcation using individualised HU threshold to compensate for the contrast-agent density. The HU of 3 non-calcified sections in the selected area was measured and averaged, and an additional 200 HU was added to this number.  - Calcification also graded as 0 (none), 1 (mild), 2 (moderate) and 3 (severe). | Semi-quantitative  Quantitative | **All Complications**  **IFA calcium volume (mm^3^):** p=0.564  **IFA calcification score (0-3):** p=0.811 | **-** |

(CFA: common femoral artery, EIA: external iliac artery, HR: hazard ratio, HU: Hounsfield units, IA: iliac artery, IFA: iliofemoral artery, IMS: iliac morphology score, MCDT: contrast-enhanced multi-detector computed tomography, OR: odds ratio.)
